# Association between cardiac autonomic control and blood pressure response to resistance training in adults with pharmacologically treated hypertension

**DOI:** 10.14814/phy2.70863

**Published:** 2026-04-06

**Authors:** Luis A. Benavides‐Roca, Germán Parra, Antonio R. Zamunér

**Affiliations:** ^1^ School of Sports Sciences and Physical Activity, Faculty of Health Universidad Santo Tomas Talca Chile; ^2^ Faculty of Education Universidad Autónoma de Chile Talca Chile; ^3^ Department of Physiology Universidad de Valencia Valencia Spain; ^4^ Laboratory of Clinical Research in Kinesiology, Department of Kinesiology and Centro de Investigación en Neuropsicología y Neurociencias Cognitivas (CINPSI Neurocog) Universidad Católica del Maule Talca Chile

**Keywords:** blood pressure, cardiovascular system, hypertension, resistance exercise

## Abstract

This study aimed to assess the acute cardiac autonomic responses to high and low intensity resistance training and their association with post exercise hypotension in adults with pharmacologically treated hypertension. This randomized clinical trial included 31 participants with pharmacologically treated hypertension. The effects of two resistance training sessions performed at high intensity (80% of 1RM) and low intensity (40% of 1RM), matched for total training volume, were compared. Blood pressure and heart rate variability were measured before and after exercise. Significance level was set at 5%. No significant interaction was observed for any of the variables (*p* > 0.05). Both intensities produced significant reductions in blood pressure and in cardiac parasympathetic indices. During the high intensity session, ΔV1% heart rate variability index was positively correlated with blood pressure reductions. During the low intensity session, low frequency was negatively correlated with blood pressure. In conclusion, both high and low intensity RT elicited acute hypotensive effects, with a more pronounced cardiac autonomic response characterized by greater vagal withdrawal observed for the low intensity training protocol. Furthermore, changes in indices related to cardiac sympathetic modulation and greater decreases in cardiac parasympathetic modulation were associated with a greater post exercise hypotensive response.

## 1 INTRODUCTION

Hypertension (HTN) is one of the most prevalent diseases worldwide and is a major predictor of cardiovascular morbidity and mortality (Campbell et al., 2023). It is considered the primary risk factor for cardiovascular diseases, with elevated systolic blood pressure (SBP) and diastolic blood pressure (DBP) being associated with increased incidence of cardiovascular events (Jones et al., 2020). This condition is also linked to an imbalance in hemodynamic regulation within the circulatory system, which contributes to elevated blood pressure (BP), therefore, the appearance of hypertension is due to a general failure of the cardiovascular system (Geng et al., 2023). Several mechanisms play a role in the pathophysiology of hypertension, including an autonomic nervous system dysfunction. Indeed, increased sympathetic activity has been observed preceding the onset of elevated BP, likely linked to central alterations in autonomic modulation (Grassi et al., 2015). In addition, increased sympathetic activity combined with reduced vagal tone represents a significant alteration in autonomic function in primary hypertension (Carthy, 2013). This is particularly important because the sympathetic nervous system (SNS) also plays a key role in mediating the baroreceptor reflex, which in turn regulates BP (Yugar et al., 2023). In this sense, although the BP is the most widely used and clinically relevant parameter for hypertension patients, it does not provide a profile of the cardiovascular autonomic function. Therefore, heart rate variability (HRV) has emerged as an accessible and complementary tool for identifying the risk of developing hypertension or monitoring disease progression, as it reflects the integrity of cardiac autonomic modulation (Carthy, 2013; Koichubekov et al., 2018; Masuo et al., 2003) and provides information on parasympathetic activity that cannot be assessed through BP alone.

Regarding the management of HTN, previous studies have shown that controlling BP solely through medication remains a challenge, as this approach effectively manages only 25%–62% of hypertensive patients (Cao et al., 2019). Therefore, implementing nonpharmacological strategies is essential to support BP regulation and improve overall health in this population (Cernota et al., 2022). In this regard, exercise has been shown to be an effective strategy for reducing negative physiological markers associated with HTN (Cernota et al., 2022). Exercise has shown to increase maximal cardiac output, peak stroke volume, diastolic filling velocity, and to reduce the rate of left ventricular remodeling (Romero‐Gómez et al., 2022). Specifically, resistance training has shown to be an effective option for this population, promoting both peripheral and central adaptations that contribute to improved cardiac autonomic control and BP regulation (De Brito et al., 2019; Mongin et al., 2022). It is noteworthy mentioning that, in patients with HTN, autonomic function is often impaired, potentially altering the physiological response to exercise. As a result, individual responses to physical activity may vary. Nevertheless, strength training remains a proven strategy to mitigate the effects of hypertension. Its benefits include post exercise hypotensive effects and increased parasympathetic dominance over sympathetic activity under low stress conditions. Evidence indicates that hypertrophy focused resistance training in individuals with prehypertension can reduce both SBP and DBP by approximately 6 mmHg, 40 min after a training session (Benavides‐Roca et al., 2025; Gambassi et al., 2020). Moreover, resistance training induces changes in HRV around 25 min post exercise, characterized by a decrease in high frequency (HF) components and a increment in low frequency (LF) components (Farinatti et al., 2021). Previous study comparing low and high‐intensity velocity‐based training showed that low‐intensity is more effective in promoting post exercise hypotension (Benavides‐Roca et al., 2025). However, the acute autonomic response to different training intensities and its relationship with BP response remains poorly understood.

Assessing acute autonomic responses to physiological stressors, such as exercise, can improve cardiovascular risk detection, especially in individuals with impaired autonomic control (Michael et al., 2017). Therefore, understanding how training intensity influences acute cardiovascular and autonomic responses could help identify exercise protocols that could be of lower risk for hypertension as well as if the postexercise hypotension could be related to the cardiac autonomic response.

Therefore, the objective of the present study was to perform a secondary analysis of a randomized controlled trial (Benavides‐Roca et al., 2025), aiming at determining the acute effects of low and high intensity resistance training on the cardiovascular response and the association between postexercise hypotension and cardiac autonomic control in individuals with hypertension treated pharmacologically.

## 2 METHODS

### 2.1 Study design

This study is a randomized controlled trial approved by the Ethics Committee of the Universidad Católica del Maule and was registered at the clinicaltrials.gov (NCT06370546).

The participants were fully informed about the research procedures, including potential risks and benefits, and all provided written informed consent prior to participation.

### 2.2 Participants

Sample size was estimated a priori using G*Power (version 3.1.9.7), based on LF/HF index data reported by Lässing et al. (2025). Assuming an effect size (Cohen's dz) of 0.56, *α* = 0.05, and 80% power, the required total sample size was 28 participants. Thirty‐one individuals with a diagnosis of primary hypertension took part in the study, aged between 30 and 60 years. The diagnosis of hypertension was confirmed by a board‐certified cardiologist according to the national guidelines for Primary or Essential Arterial Hypertension in Individuals Aged 15 Years and Older (Minsal, 2010); additionally, participants were required to have had a confirmed diagnosis for at least 1 year.

Participants were excluded if they were unable to perform the proposed exercises or if they presented cardiovascular disorders other than hypertension (such as atrial fibrillation, arrhythmias, or having a pacemaker), as well as metabolic diseases such as diabetes mellitus, hepatic, renal, or pulmonary disease.

### 2.3 Procedures

Participants attended the laboratory on three occasions. During the first visit, they were informed about the characteristics of the study and the measurement procedures. Inclusion criteria were verified, and informed consent was obtained from all participants. Anthropometric measurements were also taken using a calibrated scale (Seca model 720, Hamburg, Germany).

During subsequent visits, participants completed resistance training sessions at both high and low intensities. In each session, blood pressure and RR intervals were recorded before and immediately after exercise. To determine session intensity, opaque envelopes were prepared by a researcher not involved in data collection, and the therapist responsible for conducting the sessions opened the envelope after baseline assessments on the first day. Participants were then randomly assigned to perform either the low intensity or high intensity exercise session. Following a 1‐week washout period, participants returned to the laboratory to complete the alternate protocol.

A researcher blinded to the session was responsible for evaluations of heart rate variability and BP in the study.

Room temperature was maintained at 22°C and relative air humidity at between 40% and 60%. Participants were acquainted with the experimental protocol and were instructed to abstain from stimulants (e.g., chocolate, coffee, tea, and soft drinks) and alcoholic beverages during the 24 h preceding the visits, and to have a light meal at least 2 h before the test. To avoid any residual fatigue, subjects were asked to refrain from strenuous physical activity at least 2 days before the tests.

### 2.4 Resistance training protocol

The training protocol consisted of free weight exercises using a 7 kg barbell and additional weight plates. High intensity training was performed with 6 repetitions and 6 sets at 80% of one repetition maximum (1 RM) velocity, while low intensity training involved 12 repetitions and 6 sets at 40% of 1 RM velocity. Both interventions were designed to match in total training volume (Rodriguez‐Lopez et al., 2022), allowing for the evaluation of the impact of intensity independent of volume.

Both the low‐ and high‐intensity sessions followed the same sequence of exercises: squat, row, deadlift, and bench press. These were randomly assigned in an alternating pattern of lower‐ and upper‐body movements, and this sequence was consistently maintained throughout all training sessions. Prior to participation, all subjects received thorough instruction on proper barbell use and exercise technique to ensure safety and uniformity in execution. Each session began with a 7–10‐min warm‐up involving joint mobility exercises for both the upper and lower body. During the warm‐up, the execution velocity of each exercise was measured as part of the preparatory sets. This assessment was used to determine the appropriate load for each participant, ensuring alignment with the session's prescribed intensity. If a participant's maximal velocity exceeded the pre‐established target for a given exercise, the load was progressively increased until the velocity matched the defined threshold. Throughout the training session, each repetition was monitored in real time using mean propulsive velocity, ensuring that all exercises were performed within the intended intensity range. For the high intensity session, the mean propulsive velocities required were approximately: 0.63 ± 0.05 m/s for the squat, 1.23 ± 0.07 m/s for the row, 0.57 ± 0.05 m/s for the deadlift, and 0.50 ± 0.06 m/s for the bench press (Benavides‐Ubric et al., 2020; Hernández‐Belmonte et al., 2023). For the low intensity session, the mean velocities must be higher, as expected for lighter loads: 1.15 ± 0.09 m/s for the squat, 1.42 ± 0.10 m/s for the row, 1.02 ± 0.09 m/s for the deadlift, and 1.14 ± 0.09 m/s for the bench press (Benavides‐Ubric et al., 2020; Hernández‐Belmonte et al., 2023).

The use of velocity rather than absolute load (%1RM) helps normalize effort perception and allows a more individualized prescription approach. In our study, MPV was determined using the first repetition of each set, which corresponds to the concentric phase of maximal voluntary effort. Participants were instructed to execute each repetition with maximal intended velocity. Real‐time feedback was provided using a validated linear position transducer (ADR®, Castilla, Spain), which has been shown to offer reliable and accurate measurement of concentric bar speed (Lopez‐Torres et al., 2025). This method allows for precise control of training intensity and has been recommended in clinical populations to ensure both safety and neuromuscular stimulus.

### 2.5 Main outcomes

The main outcomes were obtained before and after each training session. Prior to the start of the session, participants were instructed to remain in a supine position for 10 min, followed by 10 min in an orthostatic (standing) position. During both time points, heart rate variability and BP were measured. BP measurements included SBP, DBP, and mean blood pressure (MBP) ([2 × DBP] + SBP ÷ 3), assessed using a digital automated device in arm (Omron HEM‐907 sphygmomanometer, Healthcare, Tokyo, Japan). BP was assessed three times with a 1‐min interval between each measurement, and the average between the three measurements was considered for the study.

#### 2.5.1 HRV analysis

On the day of the experiment, participants rested for approximately 20 min before RR interval recording. RR intervals were then recorded for 10 min in the supine position under spontaneous breathing conditions using a HR monitor (Polar V800, Polar Electro Co. Ltda. Oi, Finland) and a transmitter belt (Polar H10, Polar Electro Co. Ltda. Kempele, Finland) placed on the thoracic region at the fifth intercostal space (Giles et al., 2015). Respiratory rate was continuously monitored by visual inspection of thoracoabdominal movements to ensure stable spontaneous breathing and to identify potential extreme respiratory frequencies that could shift respiratory‐related oscillations outside conventional spectral bands. Participants with respiratory rates below 9 breaths per minute (<0.15 Hz) were excluded, as respiratory sinus arrhythmia at frequencies below 0.15 Hz may overlap with the LF band. No participant met this exclusion criterion. Importantly, no participant presented respiratory rates above 24 breaths per minute (>0.40 Hz), either before or after exercise; therefore, respiratory‐related oscillations remained within the conventional HF range (0.15–0.40 Hz). Participants were instructed to remain still and refrain from speaking during recordings to minimize artifacts.

The region of 256 consecutive beats with the greatest stability in the RR time series was selected for analysis for all participants (Catai et al., 2020; Task Force, 1996). Spectral analysis was carried out using the Kubios HRV software (V. 3.3.1, Oy, Kuopio, Finland), by applying a Fast Fourier Transform model in the previously selected RR section. The spectral components were obtained in low frequency (LF, 0.04–0.15 Hz) and high frequency bands (HF, 0.15–0.4 Hz) in absolute units (ms^2^). Normalized units were computed by dividing the absolute potency of LF or HF components by the total potency component minus the very low frequency component (0.003–0.04 Hz) and multiplying this ratio by 100. LF/HF ratio was also calculated (Task Force, 1996). Symbolic analysis was performed in order to have a better estimation of the cardiac sympathetic modulation (Porta et al., 2001). Detailed description of symbolic analysis is provided elsewhere (Porta et al., 2007). Briefly, it provides four indices: patterns without variation (0V), patterns with one variation (1V), patterns with two like variations (2LV), and patterns with two unlike variations (2UV). Previous studies (Guzzetti et al., 2000; Porta et al., 2001, 2009) have found that the 0V% index represents sympathetic cardiac autonomic modulation, 1V% and 2LV% indices represent both parasympathetic and sympathetic cardiac autonomic modulation with sympathetic predominance for the 1V% and parasympathetic predominance for the 2LV%, and 2UV% index represents parasympathetic cardiac modulation.

### 2.6 Statistical analysis

The statistical analysis was conducted using IBM SPSS Statistics for Windows, Version 22.0 (IBM Corp., New York, USA). Data are presented as mean ± standard deviation. Shapiro–Wilk test was performed to assess data normality. A two‐way repeated measures analysis of variance (ANOVA) was performed to evaluate the interaction between training intensity (high vs. low) and time (baseline vs. post) for BP and heart rate variability values. When a significant interaction was found, Bonferroni's post hoc test was applied for pairwise comparisons. Additionally, Pearson's or Spearman's correlation coefficients were conducted to examine the relationship between the blood pressure delta changes (post – pre) and HRV indices according to data normality. Correlation coefficients were interpreted as: 0.00–0.39 weak, 0.40–0.69 moderate, 0.70–0.89 strong, and ≥0.90 very strong (Schober et al., 2018). A significance level of 5% was used for all comparisons and correlations.

## 3 RESULTS

Demographic characteristics of the participants are presented in Table 1. All participants were taking antihypertensive medication, but no participant reported polypharmacy.

**TABLE 1:** Demographic characteristics of participants.

	Participants (*n* = 31)
Age (years)	45 ± 9.1
Male/female (*n*)	18/13
BMI (kg/m^2^)	29.3 ± 7.0 kg/m^2^
Medication use (%)	
Angiotensin receptor blockers (ARBs)	67.6%
Beta blockers	5.9%
Angiotensin‐converting enzyme (ACE)	14.7%
Calcium channel blockers	5.9%
Diuretics	5.9%

*Note*: Data are presented as mean ± standard deviation.

Abbreviation: BMI, body mass index.

Table 2 presents a comparison of HRV and blood BP variables following low and high intensity training. A significant intensity by time interaction was observed only for the HF component (*p* = 0.02). Additionally, a significant main effect of time was observed for several HRV parameters: mean RR (*F*_1,30_ = 38; *p* < 0.001), LF nu (*F*_1,30_ = 4; *p* = 0.04), HF nu (*F*_1,30_ = 6.6; *p* = 0.02), 0V (F_1,30_ = 11.9; *p* < 0.001), 2LV (*F*_1,30_ = 5.7; *p* = 0.02), NCI (*F*_1,30_ = 9.5; *p* = 0.01), and CI (*F*_1,30_ = 5.1; *p* = 0.03). Regarding BP variables, there was no significant difference between intensity; however, a significant effect of time was found for SBP (*F*_1,30_ = 1.6; *p* = 0.001), DBP (*F*_1,30_ = 11; *p* = <0.001), and MBP (*F*_1,30_ = 15.5; *p* = <0.001).

**TABLE 2:** Values of heart rate variability assessed before and after low‐ and high‐intensity resistance exercise sessions.

	Low intensity		High intensity		*p* Value		
	Pre	Post	Pre	Post	Time	Intensity	Time × intensity
Mean RR (ms)	859.2 ± 134.3	776.9 ± 107.1	853.1 ± 104.4	807.9 ± 109.1	<0.001	0.5	0.07
Var RR (ms^2^)	1217.1 ± 1526.3	1596.2 ± 3462.9	1052.7 ± 843.9	901.9 ± 1058.9	0.63	0.32	0.2
LF (ms^2^)	268.7 ± 292.2	200.9 ± 298.5	338.3 ± 405.6	247.7 ± 279.1	0.15	0.19	0.77
LF (nu)	54.7 ± 19.2	65.2 ± 22.5	60.2 ± 20.1	62.4 ± 21.9	0.04	0.66	0.20
HF (ms^2^)	207.1 ± 249.1	86.0 ± 121.6*	157.8 ± 159.8	139.7 ± 239.9	0.02	0.95	0.02
HF (nu)	42.8 ± 19.5	32.1 ± 20.4	36.7 ± 20.3	33.3 ± 20.4	0.02	0.29	0.24
LF/HF	2.29 ± 3.27	3.97 ± 5.32	2.80 ± 2.94	3.69 ± 4.07	0.08	0.86	0.54
0V (%)	26.1 ± 13.0	36.2 ± 18.5	28.2 ± 15.4	32.0 ± 18.3	<0.001	0.68	0.06
1V (%)	47.4 ± 5.9	43.5 ± 9.4	46.1 ± 5.7	45.3 ± 9.4	0.09	0.85	0.23
2LV (%)	9.0 ± 6.9	6.9 ± 5.6	8.1 ± 6.1	6.7 ± 4.3	0.02	0.48	0.55
2ULV (%)	17.5 ± 10.1	13.4 ± 9.6	17.7 ± 8.8	16.0 ± 10.3	0.02	0.34	0.25
SBP (mmHg)	128 ± 13	122 ± 13	125 ± 13	122 ± 12	0.001	0.37	0.21
DBP (mmHg)	84 ± 10	80 ± 7	83 ± 11	79 ± 9	<0.001	0.52	0.77
MBP (mmHg)	98 ± 12	93 ± 9	96 ± 11	93 ± 10	<0.001	0.51	0.57

*Note*: Data are presented as mean ± standard deviation.

Abbreviations: 0V, percentage zero variation RR; 1V, percentage 1 min variability; 2LV, percentage of occurrence of a two like variation pattern; 2ULV, percentage of occurrence of a two like variation pattern low frequency; DBP, diastolic blood pressure; HF, high frequency; LF, low frequency; LF/HF, relation low frequency and high frequency; MBP, mean blood pressure; Mean RR, mean interval RR; SBP, systolic blood pressure; Var RR, variation RR; VLF, very low frequency.

* *p* < 0.05 versus pre.

Table 3 shows the association between blood pressure delta changes and the indices of HRV. ΔSBP after low‐intensity velocity‐based training showed a weak positive correlation with Δ1V (*r* = 0.38, *p* = 0.04). Likewise, ΔDBP shows a weak positive correlation with the ΔLF (*r* = 0.36, *p* = 0.05). In addition, the Δ1V index presented a significant association with SBP (*r* = 0.48, *p* = 0.01), DBP (*r* = 0.61, *p* < 0.001), and MBP (*r* = 0.63, *p* < 0.001) after high‐intensity velocity‐based resistance training. Additionally, DBP shows a negative correlation with the Δ0 V index (*r* = −0.37, *p* = 0.04).

**TABLE 3:** Correlation between heart rate variability (HRV) indices delta changes and blood pressure delta changes.

	HRV indices	*r*	*p* Value
Low intensity			
ΔSBP^a^	Δ1V	0.38	0.04
ΔDBP^a^	ΔLF	0.36	0.05
High intensity			
ΔSBP^b^	Δ1V	0.48	0.01
ΔDBP^b^	Δ1V	0.61	<0.001
	Δ0V	−0.37	0.04
ΔMBP^b^	Δ1V	0.63	<0.001

Abbreviations: 0V, percentage of patterns with no variations on symbolic analysis; 1V, percentage of patterns with 1 variation on symbolic analysis; DBP, diastolic blood pressure; LF, low frequency; MBP, mean blood pressure;SBP, systolic blood pressure.

^a^ Spearman's correlation coefficient.

^b^ Pearson's correlation coefficient.

## 4 DISCUSSION

The objective of the present study was to assess the cardiac autonomic control acute response to a session of low and high‐intensity resistance training and its relationship with blood pressure changes in individuals with pharmacologically treated hypertension. The main results showed that (1) both training intensities elicited a decrease in cardiac parasympathetic modulation with a more pronounced decrease following the low intensity resistance training; (2) BP decreased following both training intensity protocols; and (3) correlation analyses revealed that increases in cardiac sympathetic modulation were associated with greater reductions in BP, suggesting a potential link between autonomic adjustments and the hypotensive effects of resistance training.

Regarding the cardiac autonomic response to low‐ and high‐intensity resistance exercise, a significant time × intensity interaction was observed in the HF component, indicating that low‐intensity training elicited a greater reduction in parasympathetic modulation compared with high‐intensity training. Although this finding may seem counterintuitive, it may be explained by the higher number of repetitions and longer time under tension required in the low‐intensity protocol to match the total training volume of the high‐intensity condition. The prolonged duration of effort likely increased metabolic stress and activation of muscle metaboreflex pathways, resulting in greater vagal withdrawal despite the lower external load. This interpretation is consistent with the findings of Vale et al. (2018), who compared two resistance exercise protocols matched for total volume but differing in intensity and repetitions (15RM vs. 6RM). In their study, only the higher‐repetition protocol significantly altered the cardiac autonomic modulation compared with a control session. Together, these findings suggest that time under tension and metabolic demand may play a more prominent role than external load per se in modulating post‐exercise autonomic responses. It is noteworthy mentioning that in the present study the training protocols were matched for total volume, which may have attenuated the expected differences in autonomic response between the two training protocols.

Regarding the correlation analysis, our results showed that patients who exhibited greater increases in the LF and 1V indices were those who presented the most pronounced hypotensive responses. LF reflects oscillatory components of heart rate variability influenced by both sympathetic and parasympathetic modulation and is strongly related to baroreflex‐mediated cardiovascular regulation (Porta et al., 2007). The 1V index derived from symbolic analysis has been associated with transitional patterns of autonomic regulation and reduced flexibility of cardiac autonomic control (Maestri et al., 2007). Similarly, the 0V index from symbolic analysis, which has been associated with cardiac sympathetic modulation (Porta et al., 2007), indicated that individuals presenting greater modulation in indices related to sympathetic predominance after exercise experienced the largest reductions in blood pressure. The use of symbolic analysis indices may provide complementary information to spectral measures, as these indices are less affected by reductions in total variability and may better capture non‐linear patterns of autonomic modulation during the post‐exercise recovery phase (Guzzetti et al., 2005). However, this finding should be interpreted with caution. The transient decrease in arterial pressure following exercise may activate baroreflex‐mediated compensatory responses, which can enhance low‐frequency oscillatory dynamics (Porta et al., 2011). Therefore, the observed associations between blood pressure reductions and indices reflecting sympathetic predominance may not necessarily indicate that sympathetic activation directly mediates post‐exercise hypotension, but rather that greater hypotensive responses trigger stronger baroreflex adjustments. Alternatively, it is possible that individuals with more preserved autonomic regulatory capacity exhibit greater cardiovascular adaptability during recovery and therefore present larger hypotensive responses to exercise. However, the present data do not allow differentiation between these mechanisms.

Overall, these findings are consistent with the notion that peripheral vascular mechanisms contribute importantly to post‐exercise hypotension. Notably, no significant associations were observed between post‐exercise hypotension and HRV indices reflecting parasympathetic modulation (e.g., HF, HFnu, and 2UV). Although increased parasympathetic activity is commonly associated with lower arterial pressure, post‐exercise hypotension is primarily attributed to peripheral vasodilatory mechanisms and reductions in systemic vascular resistance rather than to immediate vagal activation. Accordingly, the absence of a relationship between BP reductions and parasympathetic‐related indices during recovery may reflect the predominance of vascular mechanisms over cardiac autonomic adjustments in the early phase of post‐exercise recovery.

It should also be considered that participants were pharmacologically treated and presented BP values close to recommended targets at baseline, which may have limited the magnitude of post‐exercise hypotension. Nevertheless, the observation of significant acute BP reductions despite optimized baseline control underscores the potential clinical relevance of resistance exercise as an adjunct non‐pharmacological therapy.

These findings are broadly consistent with the systematic review by Brasileiro‐Santos and Santos (2017), which reported post‐resistance exercise reductions in BP accompanied by autonomic adjustments such as increased cardiac and vasomotor sympathetic modulation, reduced parasympathetic activity, and attenuation of baroreflex sensitivity. Acute increases in sympathetic modulation following exercise are likely related to catecholamine release and the associated autonomic adjustments during recovery. Similar responses have been reported by Queiroz et al. (2015), who observed post‐exercise hypotension accompanied by increases in LF following resistance training performed at moderate intensity.

Taken together, the present study demonstrates that both high‐ and low‐intensity resistance training can produce comparable acute reductions in BP and changes in cardiac autonomic modulation in individuals with hypertension. These findings suggest that both training modalities may represent safe and potentially effective non‐pharmacological strategies for BP management. Future studies should further investigate the relative contribution of autonomic and peripheral vascular mechanisms to post‐exercise hypotension and how these responses evolve with long‐term resistance training.

### 4.1 Study limitations

This study has some limitations that should be considered when interpreting the findings. First, the relatively small sample size limited the possibility of performing subgroup analyses according to sex, age, or type of antihypertensive medication, which could provide additional insight into potential differences in autonomic responses. In addition, the heterogeneous use of antihypertensive medications among participants may have influenced cardiac autonomic modulation and therefore should be considered when interpreting the HRV findings. This factor also limits the generalizability of the results to untreated hypertensive populations. Nevertheless, the randomized crossover design allowed within‐subject comparisons, which enhanced internal validity and strengthened the reliability of comparisons between exercise intensities. Another limitation is that autonomic and blood pressure responses were assessed only during the early phase of post‐exercise recovery. Although this approach allowed the characterization of early responses associated with post‐exercise hypotension, additional measurements at later time points would provide a more comprehensive understanding of the recovery dynamics. Finally, respiratory activity and continuous non‐invasive blood pressure were not instrumentally recorded. Direct measurement of respiratory patterns and blood pressure variability could have provided additional information regarding the influence of breathing on cardiac autonomic modulation, as well as a more detailed assessment of vascular sympathetic modulation and baroreflex sensitivity following resistance exercise.

## 5 CONCLUSION

In conclusion, both high and low intensity RT elicited acute hypotensive effects with a more pronounced cardiac autonomic response observed for the low intensity training protocol. Furthermore, a higher increase in the cardiac sympathetic autonomic modulation and a higher decrease in the cardiac parasympathetic modulation were associated with a greater post exercise hypotensive response.

## AUTHOR CONTRIBUTIONS

**Luis A. Benavides‐Roca:** Conceptualization; data curation; formal analysis. **Germán Parra:** Conceptualization; supervision. **Antonio R. Zamunér:** Conceptualization; formal analysis; investigation; resources; supervision.

## FUNDING INFORMATION

Agencia Nacional de Investigación y Desarrollo (ANID, Chile) CIA250016 (CPS‐RTC).

## CONFLICT OF INTEREST STATEMENT

The authors have no conflict of interest to declare.

## ETHICS STATEMENT

This study was conducted in accordance with the Declaration of Helsinki and was approved by the Ethics Committee of Universidade Católica del Maule (approval number 248–2022). Written informed consent was obtained from all participants prior to their participation in the study.

## Data Availability

Data are available from the corresponding author upon request.
